# Unveiling Magnetic Characteristics of (CoCrFeNiMn)_3_O_4_ High-Entropy Oxide: The Role of Compositional Optimization

**DOI:** 10.1021/acsomega.5c00615

**Published:** 2025-05-22

**Authors:** Samer I. Daradkeh, Mohammad M. Allaham, Tomáš Spusta, Vaclav Pouchlý, Alexandr Knápek, Pavel Tofel, Dinara Sobola

**Affiliations:** † Central European Institute of Technology, 232848 Brno University of Technology, Purkyňova 656/123, 612 00 Brno, Czech Republic; ‡ Department of Physics, Faculty of Electrical Engineering and Communication, 48274 Brno University of Technology, Technická 2848/8, 616 00 Brno, Czech Republic; § Institute of Scientific Instruments of the Czech Academy of Sciences, Královopolská 147, 612 64 Brno, Czech Republic; ∥ Academy of Sciences CR, Institute of Physics of Materials, Žižkova 22, 616 62 Brno, Czech Republic; # Institute of Materials Science and Engineering, Brno University of Technology, Technicka 2, Brno 616 69, Czech Republic

## Abstract

High-entropy oxides (HEOs) are considered promising materials in many electrical applications, especially in the fields of batteries, energy storage and conversion, and catalysis. The study material, (CoCrFeNiMn)_3_O_4_ in particular, has been shown to possess gigantic capacitance, a characteristic that emanates from its oxide precursors properties. The material was successfully prepared using the solid-state reaction method, as the material showed structural stability despite its different composition and the possibility of controlling its magnetic properties based on its composition, as the saturation value of Fe excessive addition-S2.Fe reached 52 emu/g, with it having a relatively high coercivity value, which is estimated at 1102.47 Oe. It was also observed that electron hopping between different oxidation states is based on the high-resolution X-ray photoelectron spectroscopy (XPS) results, which has a role in the magnetic properties.

## Graphical abstract

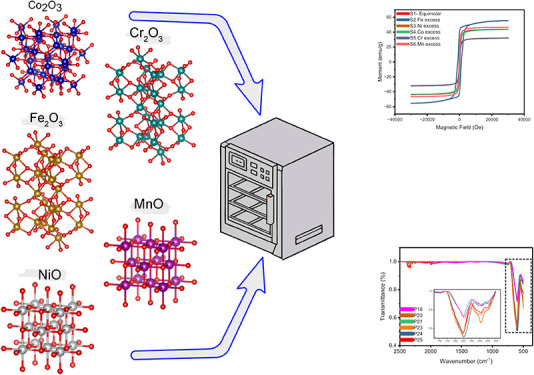

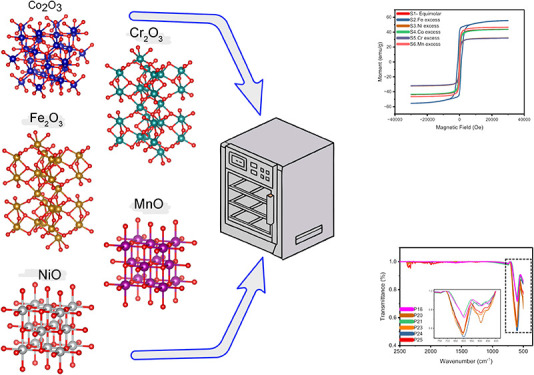

## Introduction

High-entropy oxides (HEOs) and high-entropy alloys (HEAs) represent a groundbreaking class of materials, emerging from the fundamental thermodynamic principle of configurational entropy maximization, and they can be understood through the Boltzmann equation in statistical thermodynamics. This innovative approach to materials design transcends traditional compositional strategies by deliberately introducing multiple elements into a single crystalline structure.^1^ The idea began in the 1980s and has gone on to this day.^2^ The core concept of this approach involves using various combinations of these five elements to fabricate high-entropy oxides, HEOs, or HEAs, and it proved their feasibility in a wide range of applications. The material system has been successfully explored in multiple applications, including, but not limited to, batteries,^3–5^ catalytic systems,^6^ and microwave attenuation devices,^7^ and magnetic and microwave absorption.^8^ The alloy version of the (CoCrFeNiMn)_3_O_4_ proved its versatile possible applications, for instance, excellent electromagnetic-wave absorbing performance^9^ and supercapacitive properties.^10^ As for HEO-(CoCrFeNiMn)_3_O_4_, there is relatively little research that indicates its potential use in various applications. Talluri et al. pointed out^11^ about its enhanced capacitance and its high possibility for supercapacitor electrodes. Also, it is used in electrocatalyst for methanol oxidation and oxygen evolution reactions,^12^ where it showed a specific current density of ∼335 mA.cm^–2^, and the mass activity of the catalyst is ∼110 mA.mg^–1^.

This study examines the hysteresis loop parameters, key indicators of the magnetic behavior of (CoCrFeNiMn)_3_O_4_, and explores the potential for tuning these properties through compositional modifications and attempts to make a connection between the high-resolution X-ray photoelectron spectroscopy (XPS) spectra and the magnetic properties results. The intrinsic complexity of these systems enables a high possibility of magnetic property modulation through elemental substitution and concentration adjustment. In general, the HEOs are characterized by containing different magnetic complexes, for instance, manganese oxide (MnO) and iron oxide (Fe_2_O_3_). Iron oxide, has the chemical composition of Fe^2+^(Fe^3+^)_2_(O^2–^)_4_ or Fe_2_O_3_, with cubic inverse spinel structure,^13^ characterized by having Fe ions with an antiparallel magnetic moment, but the net magnetic moment above zero. The magnetic properties that characterize Fe_2_O_3_ stem from it containing the Fe^3+^ ion, which possesses a partially filled d state. The ferric ions occupy the tetrahedral and octahedral sites, where the Fe^2+^ and Fe^3+^ occupy 1/3 tetrahedral and 2/3 octahedral sites.^14^ Owing to this structure and coordination system, it has a magnetic property, mediated by oxygen ions, along with some other properties of organic molecules.^15,16^ The Néel temperature (*T*_N_) was estimated at 966 K.^17^ The situation for MnO illustrates that mediated by O ion, type antiferromagnetic ordering arises due to superexchange interactions between the adjacent Mn cations, resulting in the antiferromagnetic coupling of Mn magnetic moments.^18^ Superexchange interaction occurs because the overlap of 3d orbitals of Mn ions with 2p orbitals of oxygen favors the antiparallel alignment of spins between neighboring Mn ions.^19^ The *T*_N_ value of MnO is estimated at approximately 118 K.^20,21^ Nickel oxide (NiO) exhibits a rock salt crystal structure characterized by a type-II antiferromagnetic configuration. This magnetic arrangement presents a unique collinear spin structure with two key magnetic alignment characteristics. Below a Néel temperature (*T*_N_) of 523 K, within each (111) crystallographic plane, Ni^2+^ cations are ferromagnetically aligned, creating a uniform magnetic orientation. Conversely, between neighboring (111) planes, the Ni^2+^ cations demonstrate antiferromagnetic alignment, resulting in an alternating magnetic moment orientation that defines the compound’s complex magnetic topology.^22,23^ Cobalt oxide (Co_3_O_4_) has a formula unit of AB_2_O_4_ and its own same spinel structure as Fe_2_O_3_, where the Co^2+^ ion occupies the tetrahedral A site and the Co^3+^ ion occupies the octahedral site of the closely packed oxygen lattice. Research indicates that Co_3_O_4_ derives its magnetic characteristics primarily from the tetrahedrally coordinated Co^2+^ ions, which possess a magnetic moment of 3.20 μ_B_. Conversely, the Co^3+^ ions are diamagnetic as a result of the substantial 3d level splitting and the pairing of six electron spins within the d orbital shell, leading to no substantial impact on the compound’s magnetic properties.^24^ The magnetic structure is characterized by the antiferromagnetic arrangement of Co^2+^ ions along the [111] crystallographic plane.^25^ These properties are pronounced at temperatures below the Néel temperature, which is estimated to be between 30 and 40 K.^26–28^ The Cr_2_O_3_ is an antiferromagnetic transition metal oxide that has a corundum structure, in which the Cr ion spin is oriented toward the *c* axis. The Cr atoms occupy octahedral coordination sites encircled by oxygen atoms, and these Cr atoms are displaced from the centrosymmetric position. This structural displacement imparts directional polarity to the coordination site, establishing a preferential orientation that influences both the electronic and magnetic properties of the oxide, which may be proper in spintronic applications.^29–31^ The Cr_2_O_3_ exhibits two magnetic exchange mechanisms: superexchange interaction and direct exchange interaction. In the superexchange configuration, oxygen atoms serve as intermediaries facilitating interactions between the magnetic moments of Cr ions. However, this superexchange mechanism contributes minimally to the overall magnetization due to relatively weak hybridization and limited overlap between oxygen electronic states and chromium t_2g_ states. Conversely, the direct exchange interaction between adjacent Cr ions demonstrates significantly stronger overlap strength compared to the superexchange pathway. This pronounced difference in interaction strengths explains the predominance of direct exchange in determining the magnetic behavior of Cr_2_O_3_. The substantial overlap between neighboring Cr ions establishes the primary magnetic coupling mechanism that governs the oxide magnetic properties and response characteristics.^29,30^

This study examines the hysteresis loop parameters, key indicators of the magnetic behavior of (CoCrFeNiMn)_3_O_4_, and explores the potential for tuning these properties through compositional modifications and attempts to make a connection between the high-resolution X-ray photoelectron spectroscopy (XPS) spectra and the magnetic properties results. The intrinsic complexity of these systems enables a high possibility of magnetic property modulation through elemental substitution and concentration adjustment. In general, the HEOs are characterized by containing different magnetic complexes, for instance, manganese oxide (MnO) and iron oxide (Fe_2_O_3_). Iron oxide, has the chemical composition of Fe^2+^(Fe^3+^)_2_(O^2–^)_4_ or Fe_2_O_3_, with cubic inverse spinel structure,^13^ characterized by having Fe ions with an antiparallel magnetic moment, but the net magnetic moment above zero. The magnetic properties that characterize Fe_2_O_3_ stem from it containing the Fe^3+^ ion, which possesses a partially filled d state. The ferric ions occupy the tetrahedral and octahedral sites, where the Fe^2+^ and Fe^3+^ occupy 1/3 tetrahedral and 2/3 octahedral sites.^14^ Owing to this structure and coordination system, it has a magnetic property, mediated by oxygen ions, along with some other properties of organic molecules.^15,16^ The Néel temperature (*T*_N_) was estimated at 966 K.^17^ The situation for MnO illustrates that mediated by O ion, type antiferromagnetic ordering arises due to superexchange interactions between the adjacent Mn cations, resulting in the antiferromagnetic coupling of Mn magnetic moments.^18^ Superexchange interaction occurs because the overlap of 3d orbitals of Mn ions with 2p orbitals of oxygen favors the antiparallel alignment of spins between neighboring Mn ions.^19^ The *T*_N_ value of MnO is estimated at approximately 118 K.^20,21^ Nickel oxide (NiO) exhibits a rock salt crystal structure characterized by a type-II antiferromagnetic configuration. This magnetic arrangement presents a unique collinear spin structure with two key magnetic alignment characteristics. Below a Néel temperature (*T*_N_) of 523 K, within each (111) crystallographic plane, Ni^2+^ cations are ferromagnetically aligned, creating a uniform magnetic orientation. Conversely, between neighboring (111) planes, the Ni^2+^ cations demonstrate antiferromagnetic alignment, resulting in an alternating magnetic moment orientation that defines the compound’s complex magnetic topology.^22,23^ Cobalt oxide (Co_3_O_4_) has a formula unit of AB_2_O_4_ and its own same spinel structure as Fe_2_O_3_, where the Co^2+^ ion occupies the tetrahedral A site and the Co^3+^ ion occupies the octahedral site of the closely packed oxygen lattice. Research indicates that Co_3_O_4_ derives its magnetic characteristics primarily from the tetrahedrally coordinated Co^2+^ ions, which possess a magnetic moment of 3.20 μ_B_. Conversely, the Co^3+^ ions are diamagnetic as a result of the substantial 3d level splitting and the pairing of six electron spins within the d orbital shell, leading to no substantial impact on the compound’s magnetic properties.^24^ The magnetic structure is characterized by the antiferromagnetic arrangement of Co^2+^ ions along the [111] crystallographic plane.^25^ These properties are pronounced at temperatures below the Néel temperature, which is estimated to be between 30 and 40 K.^26–28^ The Cr_2_O_3_ is an antiferromagnetic transition metal oxide that has a corundum structure, in which the Cr ion spin is oriented toward the *c* axis. The Cr atoms occupy octahedral coordination sites encircled by oxygen atoms, and these Cr atoms are displaced from the centrosymmetric position. This structural displacement imparts directional polarity to the coordination site, establishing a preferential orientation that influences both the electronic and magnetic properties of the oxide, which may be proper in spintronic applications.^29–31^ The Cr_2_O_3_ exhibits two magnetic exchange mechanisms: superexchange interaction and direct exchange interaction. In the superexchange configuration, oxygen atoms serve as intermediaries facilitating interactions between the magnetic moments of Cr ions. However, this superexchange mechanism contributes minimally to the overall magnetization due to relatively weak hybridization and limited overlap between oxygen electronic states and chromium t_2g_ states. Conversely, the direct exchange interaction between adjacent Cr ions demonstrates significantly stronger overlap strength compared to the superexchange pathway. This pronounced difference in interaction strengths explains the predominance of direct exchange in determining the magnetic behavior of Cr_2_O_3_. The substantial overlap between neighboring Cr ions establishes the primary magnetic coupling mechanism that governs the oxide magnetic properties and response characteristics.^29,30^

The magnetic properties of the studied high-entropy oxide materials emerge from a complex interplay of multiple mechanisms, such as coordination geometry, valence, spin state, different paths for superexchange interaction, and hybridization type and strength, demonstrating both compositional diversity and fundamental similarities in their magnetic origin, where it consists of a large number of metal–oxygen–metal couples. This intricate relationship provides a strategic framework for predictive material design and targeted property optimization.

## Results and Discussion

### Structural Investigation

#### Scanning Electron Microscopy (SEM)

The equimolarity of cation content that characterizes high-entropy alloy, as well as materials based on this idea, such as high-entropy ceramic, has not been preserved in most of the samples in order to try to improve and compare the electrical and magnetic properties by manipulating the composition. Table 1 shows the composition of the (CoCrFeNiMn)_3_O_4_ samples used in this study. Energy-dispersive X-ray spectroscopy (EDS) was used to validate the desired composition and verify the homogeneity of the material after heat treatment. The results of EDS are shown in Figure 1.

**Figure 1:**
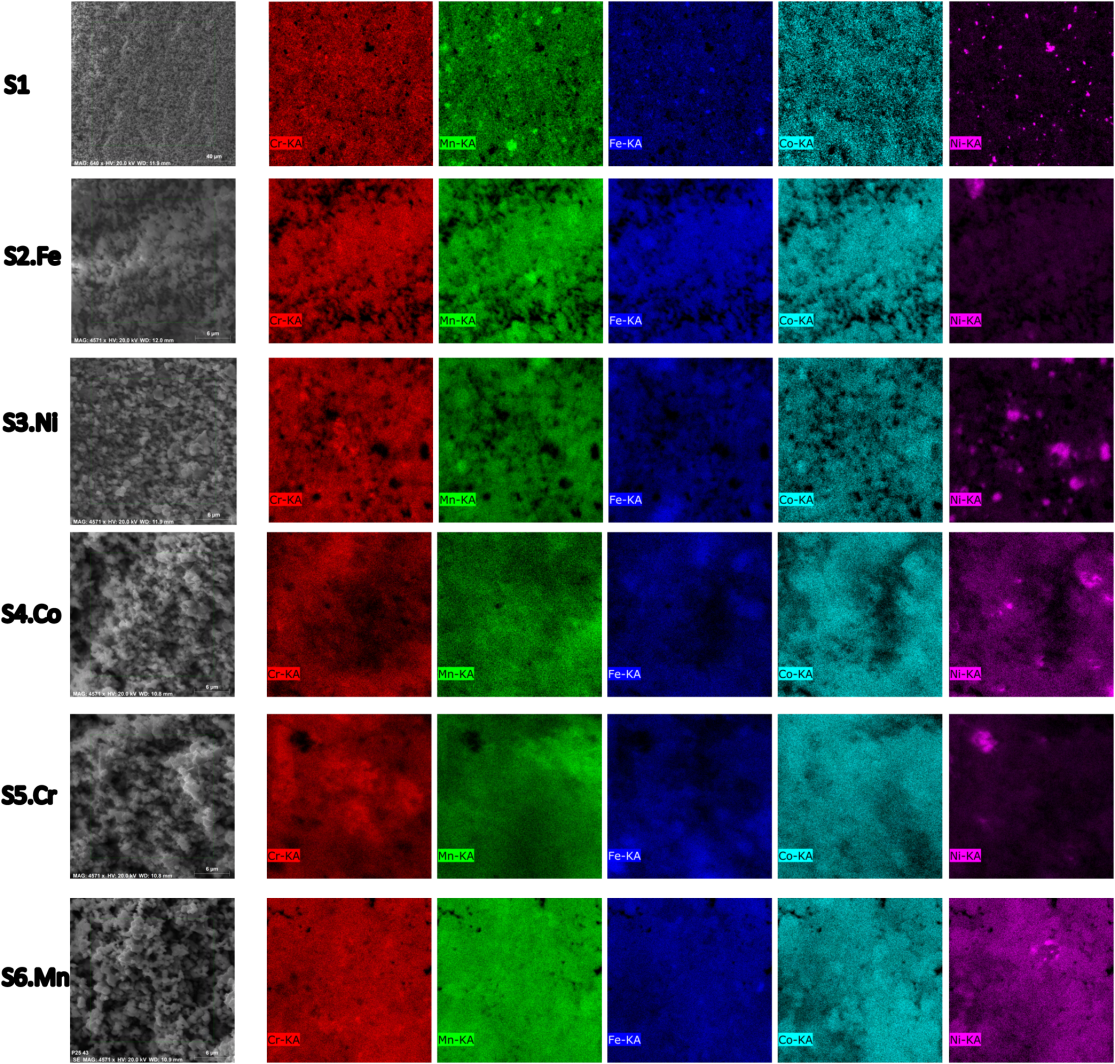
SEM images and EDS mapping of the synthesized HEOs (S1 equimolar–S6.Mn excess element) show the homogeneity degree.

**Table 1:** Obtained Compositions of the Synthesized HEOs (S1 equimolar–S6.Mn excess element) Samples via SEM/EDS

	cation element (at. %)					
sample no.	Cr	Mn	Fe	Co	Ni	excess status
S1	21.27	21.26	21.92	22.86	20.37	equimolar
S2.Fe	15.26	17.78	34.96	18.35	15.07	Fe excess
S3.Ni	20.36	20.54	21.88	21.80	37.07	Ni excess
S4.Co	16.35	18.25	16.75	34.06	14.59	Co excess
S5.Cr	33.44	18.42	16.32	16.88	14.93	Cr excess
S6.Mn	15.87	34.72	15.32	17.64	16.44	Mn excess

#### Raman Spectroscopy

Raman spectroscopy analysis was conducted on the prepared samples to confirm the structural stability, complementing the X-ray diffraction (XRD) characterization findings. Measurements were performed using a 633 nm laser excitation source to obtain the vibrational fingerprints characteristic of the material’s molecular structure. Figure 2 presents the Raman spectra collected from all samples prepared in this investigation, revealing the distinctive fingerprint patterns that correspond to the *Fd*3̅*m*:227 space group. There is a consistent and common appearance of specific Raman active modes across all samples, with variation in the intensity of some active modes. Particularly prominent was the observed intensity fluctuation at ∼650 cm^–1^, which corresponds to the characteristic inversion Raman mode, signifying the breaking of the inversion symmetry, likely attributable to an excess of transition elements. For cubic symmetry (*Fd*3̅*m* space group), according to the selection rule, there are 5 modes, which are A_1g_, E_g_, and three F_2g_.^32,33^ The A_1g_ active mode refers to the vibration of octahedral metal–oxygen bonds, whereas E_g_ and 3F_2g_ correspond to the vibration of tetrahedral metal–oxygen bonds.^12^ The bands centered at 169, 440, 508, 558, 620, and 650 cm^–1^ are assigned to F_2g_, E_g_, F_2g_, F_2g_, A_1g_, and A_1g_^′^ phonon modes, respectively. For several reasons, including sample effect, instrumental effect, and disorder effect, which contribute to the presence of an extra Raman band.^34^

**Figure 2:**
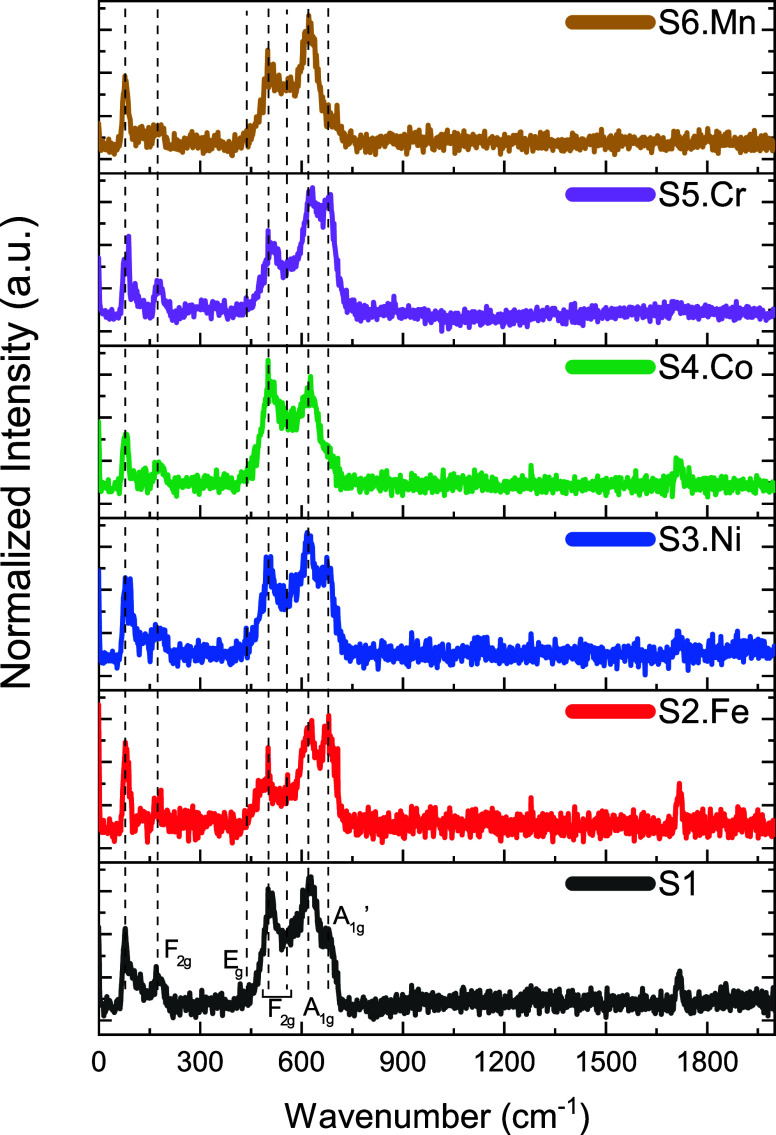
Raman spectroscopy results of—from the bottom to the top—S1 (equimolar HEO), S2.Fe (Fe excess), S3.Ni (Ni excess), S4.Co (Co excess), S5.Cr (Cr excess), and S6.Mn (Mn excess) samples.

#### X-ray Diffraction

Analyzing the results depicted in Figure 3, the crystal structure of all of the investigated samples corresponds to a single-phase cubic spinel structure with space group *Fd*3̅*m*:227, except S3.Ni. The Ni-rich sample (designated as S3.Ni) exhibited a dual-phase structure, comprising the primary cubic spinel phase (*Fd*3̅*m*:227) and a secondary monoclinic phase (*C*12/*m*1:12). Heat treatment demonstrates a significant stabilizing effect on the crystal structure, maintaining its integrity despite compositional modification. All XRD analyses were performed based on Rietveld refinement using PDXL2 software, and the results are presented in Table 2.

**Figure 3:**
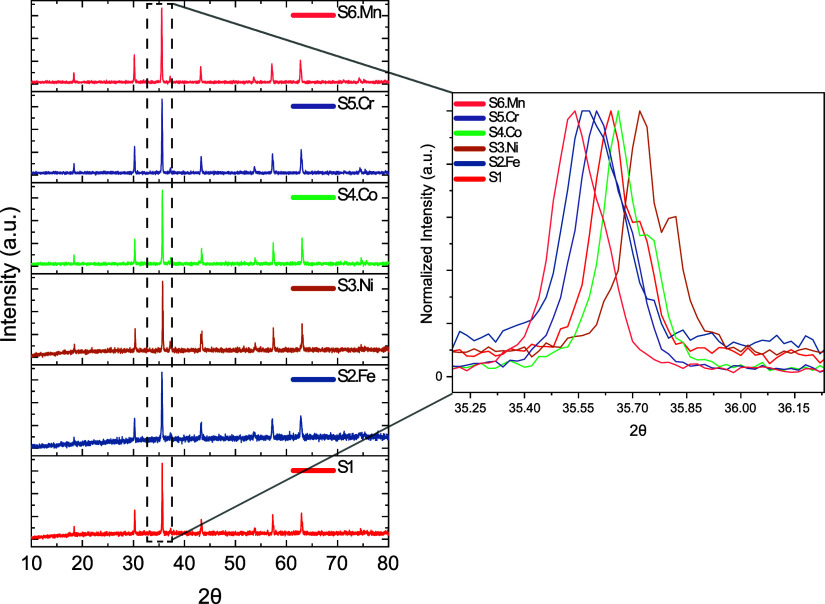
XRD diffraction pattern of synthesized HEOs (S1 equimolar–S6.Mn excess element) samples.

**Table 2:** Rietveld Refinement Factor Results (*R*-Factors *R*_wp_, *R*_p_, Goodness-of-fit (χ^2^)) of all HEO Samples (S1 to S6.Mn). Lattice Parameters (a–c) and Crystallite Size

		Rietveld refinement results							
sample	crystal structure	*R*_wp_	*R*_p_	χ^2^	*a*	*b*	*c*	crystallite size	volume (Å)
S1	*Fd*3̅*m*: 227	4.64	4.36	1.12	8.35	8.35	8.35	697	581.49
S2.Fe	*Fd*3̅*m*: 227	9.35	7.37	0.93	8.37	8.37	8.37	37	586.09
S3.Ni	*Fd*3̅*m*: 227	9.22	7.24	1.04	-	-	-	-	-
S4.Co	*Fd*3̅*m*: 227	5.61	4.38	1.58	8.33	8.33	8.33	775	578.79
S5.Cr	*Fd*3̅*m*: 227	7.02	5.67	1.53	8.35	8.35	8.35	742	583.14
S6.Mn	*Fd*3̅*m*: 227	6.19	4.78	1.48	8.11	8.11	8.11	5.1	533.2

Figure 3 reveals systematic shifts in the main diffraction peak, corresponding to variations in the *d*-spacing that correlate with compositional changes. An Expansion of the lattice is observed with excess Mn, Fe, and Cr, which relates to the peak shift to lower angles. Conversely, contraction occurs with increased Co and Ni content, which is reflected by the shift toward higher angles. These structural modifications are primarily governed by the ionic radii of the constituent elements.

#### X-ray Photoelectron Spectroscopy

Due to the surface-sensitive nature of XPS, this technique provides detailed information about surface chemistry rather than bulk composition. Therefore, XPS analysis was primarily employed to elucidate the chemical bonding states and electronic structure of the transition elements in the sample. The XPS survey spectrum confirms the presence of all constituent elements in the sample, with adventitious carbon serving as a reference for peak calibration.

The high-resolution XPS spectra (Figure 4) reveal distinct peak patterns for each transition metal, indicating multiple oxidation states within the material. Each transition element exhibits characteristic peak splitting, demonstrating the coexistence of different valence states. The high-resolution XPS spectra of Cr reveal four distinctive peaks across all samples (S1–S6.Mn) with a notable higher intensity of the Cr^3+^ peak at S3.Ni (Figure 4c)—Ni excess sample compared to its intensity in the other samples. The presence of multiple peaks suggests multiple oxidation states and complex electronic interactions within the spinel structure, which characterize the (CoCrFeNiMn)_3_O_4_ (HEO/S1 to S6.Mn samples).^35^

**Figure 4:**
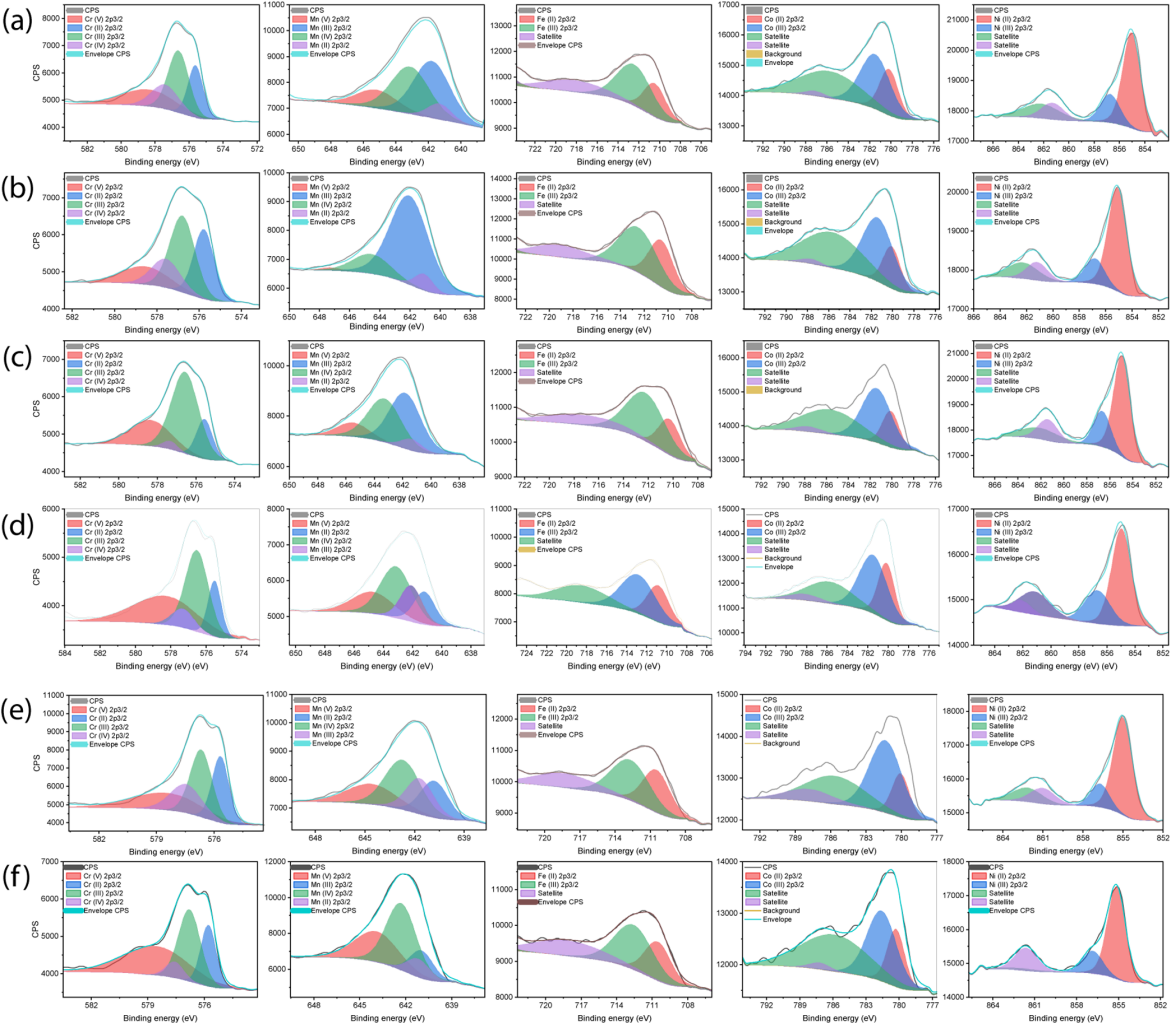
High-resolution spectra of transition elements (Cr, Mn, Fe, Co, Ni) of (a) S1, (b) S2.Fe, (c) S3.Ni, (d) S4.Co, (e) S5.Cr, (f) S6.Mn.

It can be observed that an increase in the Ni content (sample S3.Ni) is accompanied by an increase in the Cr­(II) peak. Similar behavior can be noted in S2.Fe and Mn^3+^. This phenomenon can be attributed to the intrinsic charge compensation mechanisms typical of complex multicomponent oxide systems. The presence of multiple transition metal ions facilitates coupled redox reactions, which enable local electronic charge neutrality through electron delocalization processes. The mechanism involves electron redistribution across different cation sites, promoting electronic charge transfer and valence state adjustments. This electron hopping phenomenon, facilitated by the diverse transition metal composition, allows for dynamic charge compensation without disrupting the overall structural integrity of the high-entropy oxide. The electron hopping process between the different oxidation states usually leads to a direct exchange interaction type and directly impacts the overall magnetic properties.

Comprehensive quantitative XPS analysis unveils the complex oxidation state landscape of the transition metal cations in the (CoCrFeNiMn)_3_O_4_ high-entropy oxide system. The multiple peaks for Mn, Fe, Co, and Ni demonstrate a rich electronic structure characterized by various oxidation states. The relative peak intensities of their oxidation states exhibit a systematic variation correlating with the compositional modifications, specifically the variation of the elemental excess content. Cation site occupancy and charge distribution can be dynamically adjusted to maintain charge neutrality.

### Magnetic Investigation

#### Vibrating Sample Magnetometer-VSM

Magnetic studies were conducted using the Quantum Design, VersaLab (VERSALAB) device at different temperatures (50 K to room temperature) and under the influence of a magnetic field between 3 and −3 kOe, and the results are shown in Figure 5 and Table 3.

**Figure 5:**
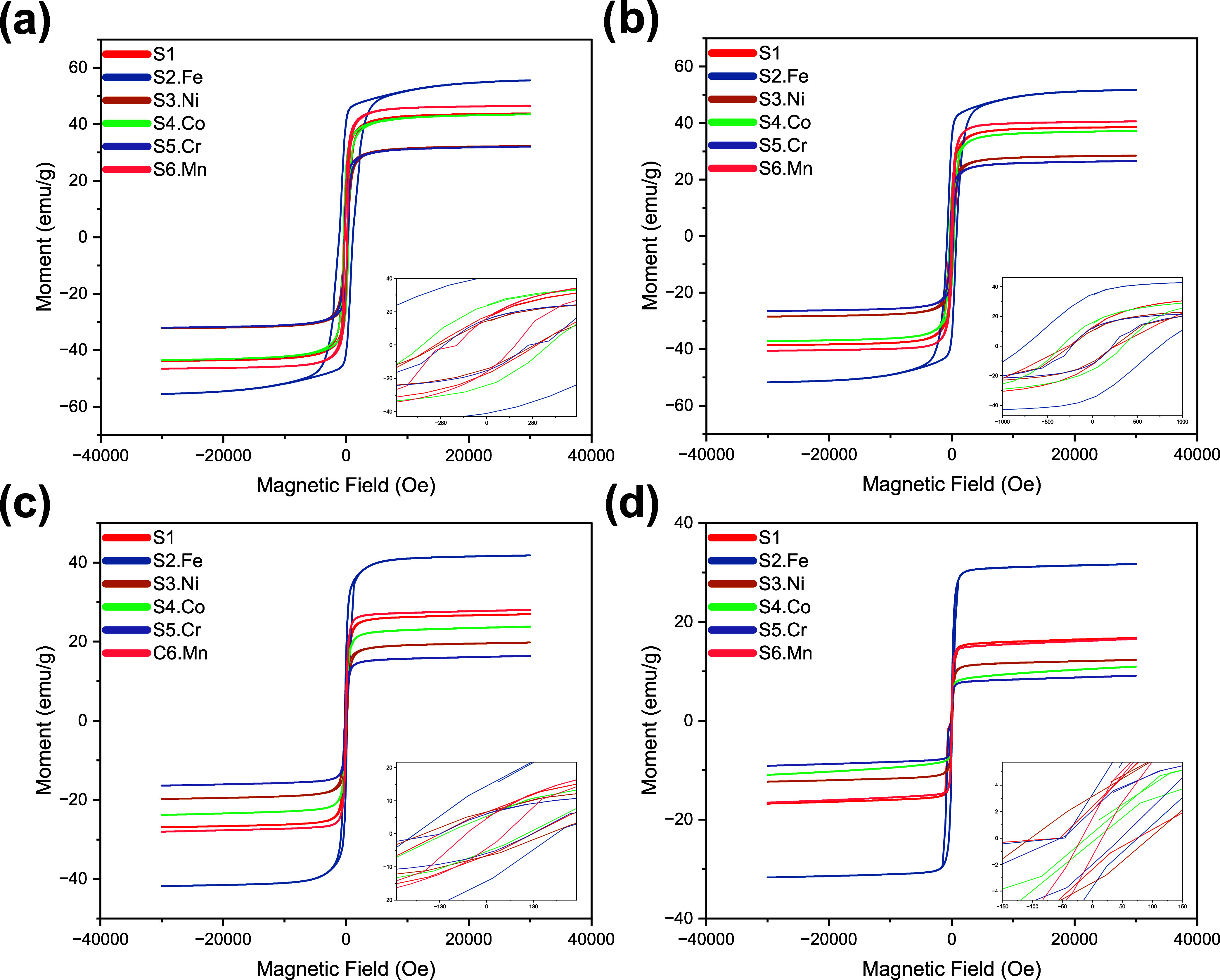
Magnetic hysteresis loops were obtained for the series of HEO samples (S1 to S6.Mn) using VSM at different degrees of measurement, revealing the magnetic behavior and domain characteristics of the (Co,Cr,Fe,Ni,Mn)_3_O_4_ multicomponent spinel system, where (a) 50, (b) 100, (c) 200, and (d) 300 K.

**Table 3:** Magnetic Parameters Identified from the Hysteresis Loops of S1 (equimolar) to S6.Mn excess element Samples at Different Measuring temperature Temperatures.

magnetic parameters	measuring temperature	S1	S2.Fe	S3.Ni	S4.Co	S5.Cr	S6.Mn
remanent (emu/g)	50	17.19	40.88	14.30	23.52	15.31	15.91
	100	12.65	34.79	10.46	15.90	11.89	10.91
	200	6.51	14.43	6.57	5.34	6.01	4.59
	300	2.38	3.29	3.15	0.37	2.07	1.50
saturation (emu/g)	50	42.98	52.57	31.49	42.31	31.10	45.72
	100	37.79	49.91	27.69	36.13	25.63	39.77
	200	26.03	40.95	18.93	22.67	15.42	26.97
	300	15.64	30.79	11.45	8.99	48.12	15.08
coercivity (Oe)	50	307.31	1102.47	292.29	381.84	261.19	176.62
	100	263.64	754.70	247.47	342.45	223.22	124.20
	200	128.36	193.13	170.42	110.70	125.0	52.66
	300	74.23	75.67	95.32	7.66	48.12	16.41

The observed relationship (Figure 5) between the magnetic moment and measurement temperature reveals the fundamental thermal dynamics of magnetic interactions in the high-entropy oxide system. This temperature-dependent magnetic behavior can be attributed to thermal agitation, which progressively disrupts the magnetic moment alignment through enhanced atomic vibrations.^36^ A recurring trend can be observed when moving from Figure 5a–d despite the measuring the temperature variation, where the highest magnetic saturation and remanent belong to the Fe-excess sample (S2.Fe). The pronounced magnetic behavior can be attributed to the high content of Fe ions that have the highest atomic spin moment, which depends on electron configuration, crystal field splitting (Δ),^37,38^ nature of the ligand,^39^ and metal–metal interaction.^40^ The magnetic properties of transition metal ions in high-entropy oxide spinels are governed by intricate crystal field interactions and site occupancy mechanisms. Specifically, the magnetic moment emerges from a complex interplay between the transition metal electronic configuration, oxidation state, and crystallographic site distribution. Iron and manganese atoms exhibit the unique capability to occupy both tetrahedral and octahedral sites within the spinel lattice.^41^ This site interchangeability enables dynamic magnetic moment modulation through charge compensation and electron delocalization processes, as the high-resolution XPS spectra show (Figure 4). The crystal field splitting value, critically determined by ligand geometry, coordination environment, and metal–ligand interactions, serves as a fundamental parameter controlling the material’s magnetic response. Consequently, the multicomponent high-entropy oxide system maintains exceptional magnetic flexibility through compositional and structural complexity, allowing for nuanced electronic and magnetic property tuning. The apparent enhancement in the Fe-excess sample (S2.Fe), suggested based on the previous results and the existence of various oxidation states and transition metal ions, is also substantiated by the presence of multiple magnetic interaction mechanisms.^42^ However, some of them are suppressed due to the nature of the HEA/HEOx systems. This does not negate the existence of some relatively short-range magnetic interaction. For example, superexchange interaction characterizes MnO_*x*_, Fe_2_O_3_, Cr_2_O_3_, and Co_3_O_4_ ^43^ mediated by O atoms. Research indicates that the superexchange interaction makes no contribution to the magnetism in ref 29 and the existence of partial covalence in the bonding in Cr_2_O_3_,^30^ a finding that may provide an explanation for the notably low magnetic response of the S5.Cr sample observed in the VSM results presented in Figure 5. The structure of the study material comprises both tetrahedral and octahedral coordination systems, so all of the cations are expected to be distributed nonpreferentially on those sites, based on the computational analyses made using the Korringa–Kohn–Rostoker (KKR) coherent potential approximation and with the help of Fe Mössbauer spectroscopy, except for the cobalt, which shows a preference for the B sublattice position.^42^ Therefore, the above data indicate the likelihood of a relatively short-range interaction that is mediated by oxygen atoms. The iron content significantly influences the magnetic properties of the HEO sample. This effect is attributed to iron ions possessing the highest Bohr magneton value among all transition elements in the composition, making them the dominant contributor to the material’s overall magnetic performance. However, it should be noted that oxygen plays a critical role in determining the magnetic properties of transition metal oxides due to its involvement in electronic interactions and orbital hybridization. So, oxygen is considered as the central of the magnetic properties in transition metal oxides. The magnetic properties of transition metal oxides are significantly influenced by the hybridization between the metal d-orbitals and oxygen p-orbitals. For instance, ferromagnetic ordering is enhanced by strong metal–oxygen hybridization, which facilitates electron delocalization and spin alignment. This has been observed in materials like manganese oxides.^44^ Oxygen’s functional significance is heightened in transition element oxides that demonstrate non-Heisenberg magnetic behavior, wherein magnetism emerges from either electron delocalization, pronounced magnetic anisotropy, or complex spin-coupling mechanisms such as the Dzyaloshinskii–Moriya interaction. In these systems, including Fe_2_O_3_,^45^ Co_3_O_4_,^25^ MnO, and Cr_2_O_3_,^29^ oxygen atoms serve as critical mediators in the magnetic exchange pathways. Conversely, some of the transition metal oxides favor Heisenberg behavior, exemplified by NiO,^45,46^ manifest magnetic properties predominantly characterized by spatially magnetic moments with interactions governed principally by isotropic exchange coupling mechanisms.

The magnetic hysteresis loop parameters exhibit characteristic temperature-dependent behaviors, as illustrated in Table 3. With increasing measurement temperature, notable changes in magnetic properties are observed. Specifically, both remanent magnetization (*M*_r_) and coercivity (*H*_c_) demonstrate an increase, reflecting the intricate relationship between the magnetic domain dynamics and thermal agitation. The temperature-dependent variation of these magnetic parameters can be attributed to the fundamental principles of thermal energy and magnetic domain wall mobility. As thermal energy increases, the enhanced molecular vibrations and lattice dynamics influence the magnetic domain structure, leading to more complex magnetic interactions. The observed increase in coercivity suggests a higher energy barrier required for magnetic domain wall movement at elevated temperatures.^47^ Conversely, saturation magnetization (*M*_s_) exhibits an inverse temperature dependence, showing a progressive increase as the measurement temperature decreases. This behavior is consistent with the fundamental magnetic properties of the material, where reduced thermal fluctuations allow for more aligned magnetic moments and consequently, a higher saturation magnetization.^48^

Another noticeable observation of saturation value difference is wherein excess Fe HEO (S2.Fe) demonstrates distinctive saturation value superiority above other investigated samples, notably commencing at 200 K. Part of the explanation of this phenomenon may be attributed to the complex interplay of magnetic phase transitions, particularly influenced by Néel temperature (*T*_N_) of the excess MnO component. It can be added to this that the *T*_N_ of Fe_2_O_3_ is considered the highest compared to the oxide precursors that were used in the preparation of HEO, which is estimated at 966 K.^17^

## Experimental Section

The method by which this substance was prepared was mentioned in previous research, which used the solid-state reaction method for its simplicity and ease.^49^ The desired material was successfully obtained with the help of heat treatment. First, the following precursor oxides were used in the synthesis process: Co_3_O_4_ (99.7%), Cr_2_O_3_ (99.0%), Fe_2_O_3_ (99.0%), NiO (99.0%), and MnO (99.0%) were proportionally weighed to establish the (CoCrFeNiMn)_3_O_4_ in the final product. The oxide mixture was subjected to wet milling in ethanol with a 5 mm zirconia grinding media. The milling parameter was optimized to 300 rpm for 60 min, which effectively enhanced the mixture’s compositional uniformity. Following the milling process, the suspension was dried, mechanically disaggregated, and subsequently sieved through a 100 μm mesh screen to obtain the initial ceramic precursor powder. The powders were calcined in an air atmosphere at 1000 °C for 20 h, with heating and cooling rates set at 5 °C/min, to achieve structural stabilization and obtain a single phase. Scanning electron microscopy/energy-dispersive X-ray spectroscopy TESCAN LYRA3 (SEM/EDS) was employed to study the composition and morphology of the samples. The X-ray diffraction (XRD) studies were employed using RIGAKU3 diffractometer (Cu as X-ray source λ = 1.5406 Å) for all samples on angle range from 10–80° to analyze the phase structure. X-ray photoelectron spectroscopy (XPS) analysis was performed using an Axis Supra (KRATOS) system to investigate the surface chemistry of the samples to depths of 5–10 nm. The system, equipped with a delay line detector photoelectron spectrometer, employed monochromatic Al Kα radiation (12 mA, 12 kV) in hybrid spectroscopy mode. This configuration, combining electrostatic and magnetic lenses, provided enhanced sensitivity over an analysis area of approximately 700 m × 300 m. XPS spectra were obtained in a binding energy range of 0–1200 eV using an analyzer pass energy of 80 eV with a step size of 0.1 eV for a high-resolution scan. Spectral analysis and peak deconvolution were conducted by using CasaXPS software. For studying the magnetic properties, a cryogenic-free vibrating sample magnetometer (VSM)­Quantum Design, VersaLab (VERSALAB) was used at a magnetic field ranging between 3 to −3 kOe.
